# Dissecting Distinct Roles of NEDDylation E1 Ligase Heterodimer APPBP1 and UBA3 Reveals Potential Evolution Process for Activation of Ubiquitin-related Pathways

**DOI:** 10.1038/s41598-018-28214-2

**Published:** 2018-07-04

**Authors:** Harbani Kaur Malik-Chaudhry, Zied Gaieb, Amanda Saavedra, Michael Reyes, Raphael Kung, Frank Le, Dimitrios Morikis, Jiayu Liao

**Affiliations:** 1 Department of Bioengineering, Center for Bioengineering Research, Bourns College of Engineering, University of California at Riverside, 900 University Avenue, Riverside, CA 92521 USA; 2 Institute for Integrative Genome Biology, University of California at Riverside, 900 University Avenue, Riverside, CA 92521 USA

## Abstract

Despite the similar enzyme cascade in the Ubiquitin and Ubiquitin-like peptide(Ubl) conjugation, the involvement of single or heterodimer E1 activating enzyme has been a mystery. Here, by using a quantitative F**ö**rster Resonance Energy Transfer (FRET) technology, aided with Analysis of Electrostatic Similarities Of Proteins (AESOP) computational framework, we elucidate in detail the functional properties of each subunit of the E1 heterodimer activating-enzyme for NEDD8, UBA3 and APPBP1. In contrast to SUMO activation, which requires both subunits of its E1 heterodimer AOS1-Uba2 for its activation, NEDD8 activation requires only one of two E1 subunits, UBA3. The other subunit, APPBP1, only contributes by accelerating the activation reaction rate. This discovery implies that APPBP1 functions mainly as a scaffold protein to enhance molecular interactions and facilitate catalytic reaction. These findings for the first time reveal critical new mechanisms and a potential evolutionary pathway for Ubl activations. Furthermore, this quantitative FRET approach can be used for other general biochemical pathway analysis in a dynamic mode.

## Introduction

Ubiquitin and Ubls are peptides that are conjugated to various target proteins to either lead the targeted protein to degradation or changes of activities *in vivo*, and their dysregulations often leads to various diseases, such as cancers or neurodegenerative diseases^1–3^. The conjugation of these peptides are conducted through a conserved but distinct enzymatic cascade involving the activating enzyme E1, the conjugation enzyme E2, and the ligases E3^1–5^. In general, each Ubl has one or two E1s, one or multiple E2s and numerous E3s^1,6–8^. These enzymes are modular in nature, with conserved domains that catalyze similar reactions in different pathways, but are specific for their own Ubl^9^. The Ubl conjugation begins with an activation enzyme (E1) that catalyzes ATP-dependent activation of the Ubls by adenylation at their C-terminal tails. E1 for ubiquitin is a single protein, Uba1, while E1s for NEDD8 and SUMO are heterodimers: APPBP1-UBA3 for NEDD8, and AOS1-Uba2 for SUMO1, respectively. The activated Ubl is transferred to a cysteine of the conjugating enzyme E2 (many in the case of ubiquitin; Ubc12 and Ube2F in the case of NEDD8; and Ubc9 in the case of SUMO) to form a thioester linkage^7,10^. Transfer of activated Ubl from E1 to E2 yields the Ubl-E2 intermediate, which serves as a donor in the final reaction of NEDDylation of the targets with the aid of E3 ligase *in vivo*. Ubiquitin and Ubl E1s appear to have an evolutionary link with the bacterial biosynthetic enzymes, MoeB and ThiF, which catalyze adenylation of C-termini of MoaD and ThiS, structural homologs of ubiquitin and Ubls^9^.

Since either one or two subunits of E1 activating enzymes are involved in the complex Ubl conjugation cascade in humans and mammals, it is interesting to understand the molecular mechanisms that shuffle different Ubls. A considerable amount of research using conventional biochemical and biophysical tools such as Co-immunoprecipitation and X-ray crystallography have shed light on the biochemical mechanisms and protein interactions in the Ubl enzymatic conjugation cascade^6–8,10–14^. For NEDD8 activation, although activating domain is located in UBA3, both subunits of E1 activating enzyme, APPBP1 and UBA3, form a complex and function as one enzyme^8,10,12^. The APPBP1-UBA3 heterodimer functions as E1 and catalyzes an adenylation reaction to produce an adenylated NEDD8 intermediate with ATP at its C-terminal Gly. Adenylated NEDD8 is transferred to UBA3 to form a high-energy thioester intermediate, which is subsequently transferred to Ubc12 (E2) to produce the thioester intermediate again^15^. However, there are still significant gaps in the knowledge of the respective conjugation cascades, such as the activation mechanisms for each Ubl, binding affinities between enzymes and Ubls or proteins of the multi-enzyme complexes, and intermediates dynamics. These understandings provide not only mechanistic understandings of these conjugation pathways but also critical evidences for future drug discovery. A detailed and quantitative delineation of the Ubl pathways requires the development of new technologies.

We previously developed novel highly sensitive and quantitative FRET tools to determine the affinities of protein-protein interactions and the dynamics and specificities of enzymatic reactions in the SUMO^16^, the ubiquitin^17^, and the NEDD8^18^ enzymatic cascades. Here we report d, for the first time, an unanticipated discovery that, one subunit of NEDD8 E1 activating enzyme, APPBP1, can be dispensable in NEDD8 activation, in contrast to that of SUMOylation, which both subunits of E1 activating enzyme, Aos1 and Uba2 are absolutely required for SUMO activation by using the quantitative FRET technology. Aided with Analysis of Electrostatic Similarities Of Proteins (AESOP) computational framework, we also systematically dissected the reaction dynamics overtime, reaction intermediates, and both covalent and non-covalent molecular interactions of the three players, namely NEDD8, APPBP1 and UBA3, during NEDD8 activation in real time. Our studies provide novel mechanistic insights into E1 enzyme activation and shed light on the evolutionary pathway of the Ubl conjugation cascades at molecular level.

## Methods

### DNA constructs

The open reading frames of CyPet and YPet were amplified with forward and reverse primers containing NheI and SalI sites, respectively, and the open reading frames of NEDD8, APPBP1, UBA3 and Ubc12 were amplified by PCR with forward and reverse primers containing SalI and NotI sites, respectively. All PCR products were cloned into the pCRII-TOPO vector (Invitrogen). After their sequences were confirmed, the genes encoding NEDD8 and NEDD8 ligases were ligated into linearized pCRII-CyPet or pCRII-YPet plasmids. After the sequences were confirmed, the cDNAs encoding fusion proteins were digested with NheI/NotI and cloned into the NheI/NotI sites of the pET28(b) vector (Novagen). To get non-fluorescent tagged proteins, the open reading frames of NEDD8 and NEDD8 were extracted by SalI/NotI digestion and cloned into the SalI/NotI sites of the pET28(b) vector. CyPet-linker3-NEDD8 was cloned as described^18^. We used full-length cDNA for all NEDD8, APPBP1, UBA3 and Ubc12. Mutant forms of APPBP1and UBA3 were created by PCR site-directed mutagenesis and cloned into the pET28(b) vector, as above. The mutations in APPBP1 and UBA3 for the NEDD8 interface were selected by closely investigating the X-ray crystal structure (1R4N)^19^, and mutations in APPBP1 for UBA3 interface and one mutation in UBA3 and NEDD8 were selected based on integrated Analysis of Electrostatic Similarities of Proteins (AESOP) analysis (see below).

### Computational alanine scan and electrostatic analysis

We selected mutations in the protein interfaces based on electrostatic calculations. Calculations were performed using the R version of the computational framework AESOP (Analysis of Electrostatic Similarities Of Proteins) to delineate the role of charged amino acids in binding^20–22^. This approach is useful for understanding the mechanism of protein-protein interactions, by performing computational alanine scans as a means of perturbation to quantitatively assess the impact of each mutation to the stability of protein complexes. The approach provides insights on the role of electrostatics in the formation of complexes between highly charged proteins, and predicts the effects of mutations on protein-protein interactions. Because the net charge of APPBP1, UBA3, and NEDD8 is −18, −9, and 0, respectively, we reasoned that elucidation of the effects of charged amino acids in the stability of the APPBP1-UBA3-NEDD8 complex and pairwise complexes APPBP1-UBA3, UBA3-NEDD8, and APPBP1-NEDD8, is essential to understand the mechanism of the specific protein-protein interactions.

AESOP performs two classes of analyses for the family of alanine scan mutants and parent (wild type) protein: clustering of proteins based on the similarity of spatial distributions of electrostatic potentials, and electrostatic free energy calculations taking into account Coulombic and desolvation effects. Electrostatic potentials are numerically calculated on a grid surrounding the protein or protein complex, according to the Poisson-Boltzmann method^23^, and are used to: (i) calculate a matrix of pairwise electrostatic potential similarities and perform hierarchical clustering, and (ii) calculate electrostatic free energies of association. AESOP analysis is based on the three-dimensional structure of the protein complex at atomic resolution. The output of AESOP is hierarchical clustering dendrograms and free energy graphs. Pairwise similarities of electrostatic potentials are calculated according to the electrostatic similarity distance (ESD) equation^22^,

1 $$ESD=\frac{1}{N}{\sum }_{i,j,k}\frac{|{\phi }_{B}(i,j,k)-{\phi }_{A}(i,j,k)|}{\max (|{\phi }_{B}(i,j,k),{\phi }_{A}(i,j,k)|)}$$

where ${\phi }_{A}$ and ${\phi }_{B}$ refer to electrostatic potential of proteins A and B, respectively, at grid point (i, j, k), and N represents the total number of grid points at which electrostatic potential has been calculated. An (n + 1) × (n + 1) ESD matrix was generated, where n + 1 corresponds to the number of mutants (n) and the parent protein. An ESD value of 0 denotes identical electrostatic potentials, and as the ESD value increases, the dissimilarity in electrostatic potential increases. A standard algorithm of hierarchical clustering with average linkage was used to generate electrostatic similarity clustering dendrograms for the family of n + 1 proteins. Electrostatic free energies of association were calculated according to a thermodynamic cycle, which incorporates Coulombic and desolvation effects in the calculation of electrostatic free energies of association^22^. The thermodynamic cycle decomposes the association process into a reference association process and a solvated association process, and two solvation processes for the reactant proteins and the product protein complex. The solvated process corresponds to association in the state of interest, the solution state, using different dielectric coefficients for the protein and solvent and in the presence of ionic strength. The reference process corresponds to association driven by Coulomb’s law, using a single dielectric constant (that of the protein) and no ionic strength. The solvation processes represent the transfer of charges from the uniform-dielectric reference state to the dual-dielectric solution state, given by the equations

2 $${\rm{\Delta }}{G}^{solution}-{\rm{\Delta }}{G}^{reference}={\rm{\Delta }}{G}_{AB}^{solvation}-{\rm{\Delta }}{G}_{A}^{solvation}-{\rm{\Delta }}{G}_{B}^{solvation}={\rm{\Delta }}{\rm{\Delta }}{G}^{solvation}\,$$

3 $${\rm{\Delta }}{G}^{solution}={\rm{\Delta }}{\rm{\Delta }}{G}^{solvation}+{\rm{\Delta }}{G}^{reference}$$

where AB refers to the protein complex, e.g. APPBP1-UBA3 or UBA3-NEDD8 (Fig. 6). ${\rm{\Delta }}{G}^{solution}$ and ${\rm{\Delta }}{\rm{\Delta }}{G}^{solvation}$ were calculated using the linearized Poisson-Boltzman equation, whereas ${\rm{\Delta }}{G}^{reference}$ was calculated using Coulomb’s equation.

The coordinates of the APPBP1-UBA3-NEDD8 complex were obtained from the Protein Data Bank (PDB code: 1R4N). The program PDB2PQR^24^ was used to add atomic radii and partial charges to the atomic coordinate file using the PARSE force field^25^ for electrostatic potential calculations with the Adaptive Poisson-Boltzmann Solver (APBS)^23^, or the stand-alone program COULOMB supplied by the APBS suite.

The molecular (dielectric boundary) and ion accessibility surfaces were determined using spherical probes and radii set to 1.4 and 2.0 Å, respectively. The APBS calculations were performed on a grid consisting of 225 × 225 × 225 grid points with grid dimensions of 204 Å × 202 Å × 154 Å. For the clustering analysis and the calculation of electrostatic potentials in solution, the dielectric coefficients for the solvent and protein were set to 78.54 and 20, respectively^26^, and ionic strength was set to 150 mM. For the calculation of electrostatic potentials in the reference state the dielectric coefficient was set to 20 for both solvent and protein^21^, and the ionic strength was set to 0 mM. All molecular graphics and analyses were performed with the UCSF Chimera package^27^.

### Protein expression and purification

BL21(DE3) *Escherichia coli* cells were transformed with pET28 vectors encoding NEDD8, APPBP1, UBA3, Ubc12, their mutants, and fluorescent protein-labeled versions. The transformed bacteria cells were plated on LB agar plates containing 50 μg/mL kanamycin, and single colony cultures were inoculated in 2xYT medium. The expression of poly-histidine-tagged recombinant proteins was induced with 0.2 mM IPTG at 25 °C overnight. Bacterial cells were collected by centrifugation at 8,000 rpm 5 min, resuspended in buffer containing 20 mM Tris-HCl, pH 7.5, 500 mM NaCl and 5 mM imidazole, and sonicated with an ultrasonic liquid processor (Misonix, Farmingdale, NY). Cell lysate containing recombinant proteins was cleared by centrifugation at 35,000 g for 30 min. The recombinant proteins were then bound to Ni^2+^-NTA agarose beads (QIAGEN, Valencia, CA) and eluted with buffer containing 20 mM Tris-HCl, pH 7.5, 200 mM NaCl, and 150 mM imidazole. Then the proteins were dialyzed into a buffer of 20 mM Tris-HCl, pH 7.5, 50 mM NaCl, and 1 mM DTT. SDS-PAGE and Coomassie blue staining confirmed the purity of the proteins. Protein concentrations were determined using Coomassie Plus Protein Assay (Pierce) and concentrations of fluorescent-tagged proteins were determined using fluorescent protein concentration standard curves.

### FRET measurement

**Figure 1:**
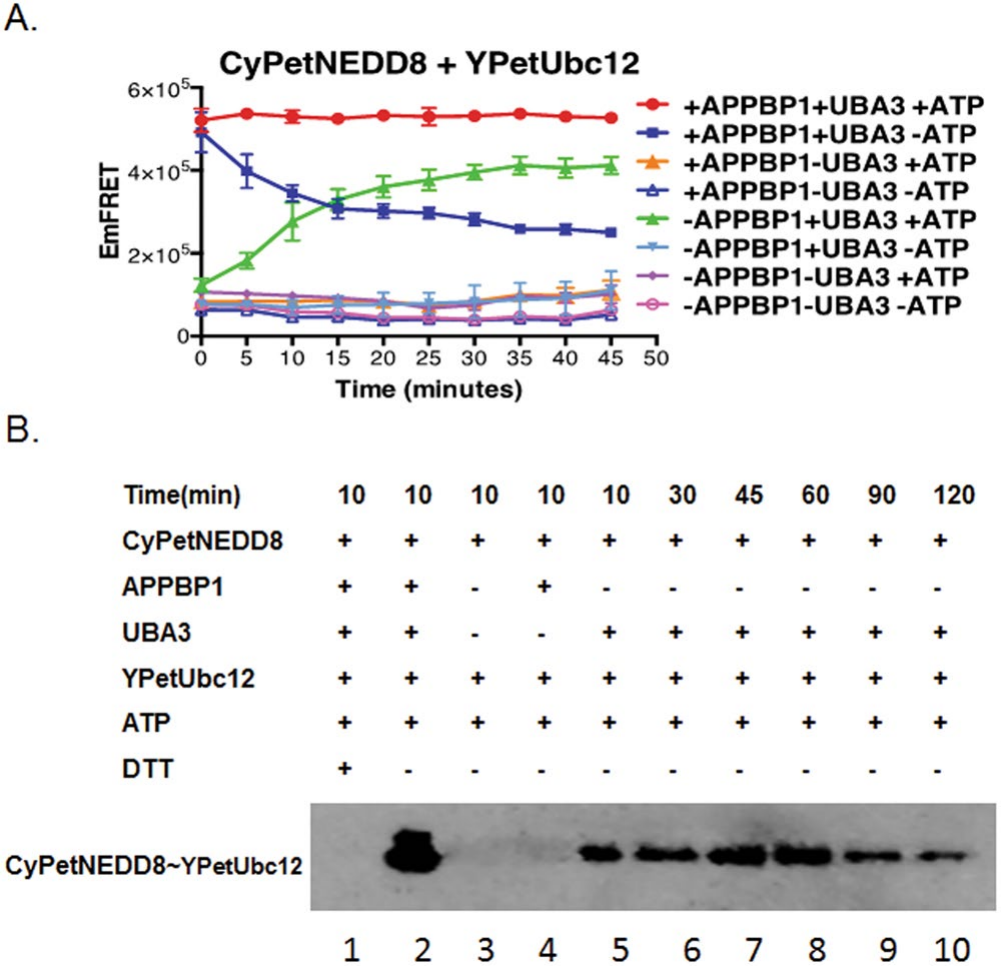
Subunit of E1, Uba3, alone is sufficient for NEDD8 activation. (**A**) FRET-based protein assay for determining the E1 requirement of APPBP1 and UBA3 for NEDD8 activation and conjugation to Ubc12(data are represented as mean +/− SD). The Time in x-axis represents the time of components mixing time. (**B**) Neddylation assay by Western-blot analysis. The conjugation of NEED8 to Ubc12 was performed with or without APPBP1 at different time points and blotted with anti-NEEd8 antibody.

After the proteins genetically labeled with CyPet or YPet were mixed in the presence or absence of other protein members or cofactors, their fluorescence was determined by the fluorescent plate reader Flexstation II^384^ (Molecular Devices) in a 384-well plate. The emission intensities at three wavelengths were collected: 475 and 530 nm at an excitation wavelength of 414, and 530 nm at an excitation wavelength of 475 nm. After the emission intensities were corrected by subtraction of background fluorescence from the plate, the EmFRET for the reactions were calculated and compared. To use FRET to monitor the change of protein-protein interaction status, we defined the EmFRET (*Em*_*FRET*_ = *Em*_*total*_ − *Cypet*_*(cont)*_ − *Ypet*_*(cont*)_), (Fig. 1)^16,20^. The FRET results were then displayed using Prism 6 (GraphPad Software, Inc.).

### Protein interaction and *in vitro* NEDDylation assay

All reagents were purchased from Research Products International Corp unless otherwise specified. For the respective FRET assay, 1 μM CyPetNEDD8 was mixed with 1 μM YPetUbc12, in presence or absence of APPBP1, APPBP1(mutants), UBA3 and UBA3(mutants)(1 μM), in NEDDylation buffer (50 mM Tris-HCl, pH 7.4, 50 mM NaCl, 10 mM MgCl_2_, 1 mM DTT). Samples were incubated at 37 °C and fluorescence emission was monitored right after buffer with or without ATP was added (2 mM final concentration).

### Western blot assay for NEDD8 conjugation to Ubc12

For the western blot assay, 0.25 μM CyPetNEDD8, 0.1 μM APPBP1, 0.1 μM UBA3 and 0.25 μM YPetUbc12 were mixed in a buffered solution containing 50 mM Tris-HCl, pH 7.4, 50 mM NaCl, 1 mM DTT, 10 mM MgCl_2_ and 1 mM ATP with a total volume of 50 μL. Samples were incubated at 37 °C for 10–120 min. The reactions were terminated by addition of non-reducing 4 M urea/SDS-gel loading buffer. After they were separated by 10% SDS-PAGE and transferred to a nitrocellulose membrane, the NEDD8 proteins were recognized by incubation with mouse anti-NEDD8 antibody (Santa Cruz Biotechnology) and then HRP-labeled goat anti-mouse IgG antibody (Sigma Aldrich). The chemiluminescence was developed using the Supersignal® West Dura Extended Duration Substrate (Thermo Scientific) and two different exposure times, 60 minutes for CyPetNEDD8~YPetUbc12 and 5 min for CyPetNEDD8, and captured by the Biospectrum® imaging system (UVP, LLC).

### Availability of materials and data

The materials and protocols are available to public. The request should be addressed to JL (jiayu.liao@ucr.edu).

### Background

The conjugation of NEDD8, an Ubiquitin-like peptides, requires an enzymatic cascade, including E1, E2 and E3.

### Results

The roles of each subunit of E1 heterodimer are dissected using quantitative FRET assay.

### Conclusion

Only one subunit of E1 heterodimer, UBA3, is absolutely required for NEDD8 activation.

### Significance

Dissecting distinct roles of E1 heterodimer is critical for understanding Ubl activation in general.

## Results

We applied our a high-sensitive and quantitative FRET-based assay to dissect the mechanisms underlying NEDD8 activation and its subsequent conjugation cascade in detail (Supplement Methods and supplement Fig. 1).

*APPBP1 is not required for NEDD8 activation-* We first investigated the NEDD8 activation by examining formation of the NEDD8/Ubc12 thioester intermediate in the presence or the absence of the E1 heterodimer or ATP. We used a CyPet-NEDD8 and YPet-Ubc12 FRET pair, which, when the thioester intermediate is formed, should produce a high Em_FRET_ signal. Indeed, in the presence of APPBP1, UBA3 and ATP, conjugation of CyPet-NEDD8 to YPet-Ubc12 led to a strong and rapid FRET signal (Red in Fig. 1A), which was stable for at least 50 minutes.

We next assessed the impact of removal of one subunit of E1 heterodimer on NEDD8 activation. In the absence of UBA3, but presence of APPBP1, neither the covalent nor the non-covalent intermediates could form (Orange triangle and Blue triangle Fig. 1A), suggesting the essential function of UBA3 as an activating enzyme. Interestingly, in the absence of ATP and presence of E1 heterodimer, the Em_FRET_ signal was high initially but decreased rapidly, suggesting that CyPet-NEDD8 and YPet-Ubc12 formed an unstable complex (Solid blue squares in Fig. 1A), which may represent transient non-covalent interactions that dissociate quickly. However, in the absence of APPBP1, but presence of UBA3 and ATP, CyPet-NEDD8 could conjugate to YPet-Ubc12, albeit with a slower reaction kinetics and a moderate reduction in activity (Green triangle in Fig. 1A).

**Figure 2:**
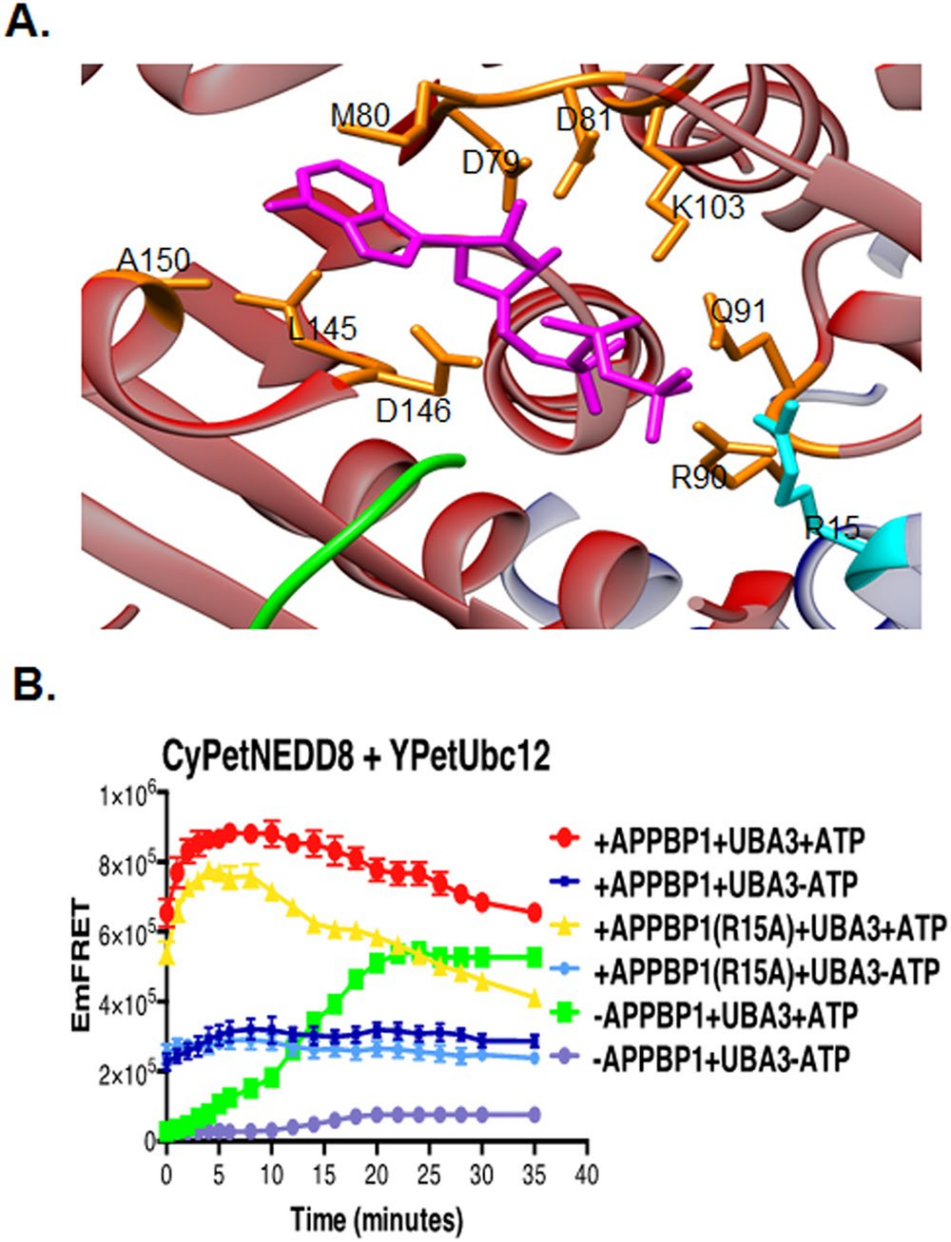
APPBP1-ATP binding activity contributes, but is not required for NEDD8 activation. (**A**) Close-up view of the ATP-binding pocket in E1 heterodimer of NEDD8 (PDB ID: 1R4N), UBA3 shown in red, APPBP1 in blue, NEDD8 in green, ATP in magenta, residue from UBA3 participating in ATP binding pocket in orange and from APPBP1 in cyan (parts of APPBP1 and UBA3 are hidden for clarity). (**B**) APPBP1’s ATP-binding residue (Arg15) is not critical for NEDD8 conjugation to Ubc12 (data are represented as mean +/− SD).

We then confirmed this intriguing discovery using Western blotting analysis, in which the covalent thioester intermediate of CyPet-NEDD8/YPet-Ubc12 was observed and could be readily dissociated by DTT (lanes 1 and 2 in Fig. 1B). In the absence of APPBP1, the covalent thioester intermediate could also form (lanes 5–10 in Fig. 1B), in consistent with the FRET results. Thus, based on our FRET-based results and western blot, we confirm APPBP1 is not required for formation of the covalent thioester intermediate of the NEDD8-Ubc12 complex. Finally, removal of any two factors in combination or three factors altogether all severely impaired NEDD8 conjugation to Ubc12, as show in the Fig. 1A. *ATP, but not NEDD8, binding activity of APPBP1 accelerates, but is not required for NEDD8 activation-*The weak effect of APPBP1 removal on NEDD8 activation was surprising and suggested that APPBP1 does not act as an activating enzyme, as previously recognized, and instead functions to accelerate the conjugation reaction. The crystal structure of APPBP1 with UBA3, NEDD8 and ATP shows that APPBP1 interacts with all the three components^19^, prompting us to investigate whether APPBP1 serves as a scaffold protein to facilitate the molecular interactions, thus enhancing the enzymatic reaction. We first examined the role of APPBP1 binding to the ATP binding pocket of the APPBP1-UBA3-NEDD8 protein complex (Fig. 2A)^19^. Of the 15 residues that interact with ATP, one residue, Arg15, is from APPBP1, while the rest are from UBA3^19^ (Fig. 2A). We mutated Arg15 of APPBP1 to Ala (APPBP1-R15A), which resulted in slower activation kinetics, without significantly impacting NEDD8 conjugation to Ubc12 (comparing the red and yellow curves in Fig. 2B). Interestingly, with the presence of the mutant APPBP1-R15A protein and in the absence of ATP, NEDD8 failed to form a stable complex with Ubc12, similar to wild type APPBP1 [compare the Blue (wild-type APPBP1) and the Light blue curve (mutant APPBP1) in Fig. 2B), and instead formed a low-affinity NEDD8-Ubc12 complex (comparing the Blue and the Light blue curve in Fig. 2B). These discoveries suggest that APPBP1 binding to ATP is not essential for the catalytic activity of ATP hydrolysis but instead accelerates the activation reaction.

**Figure 3:**
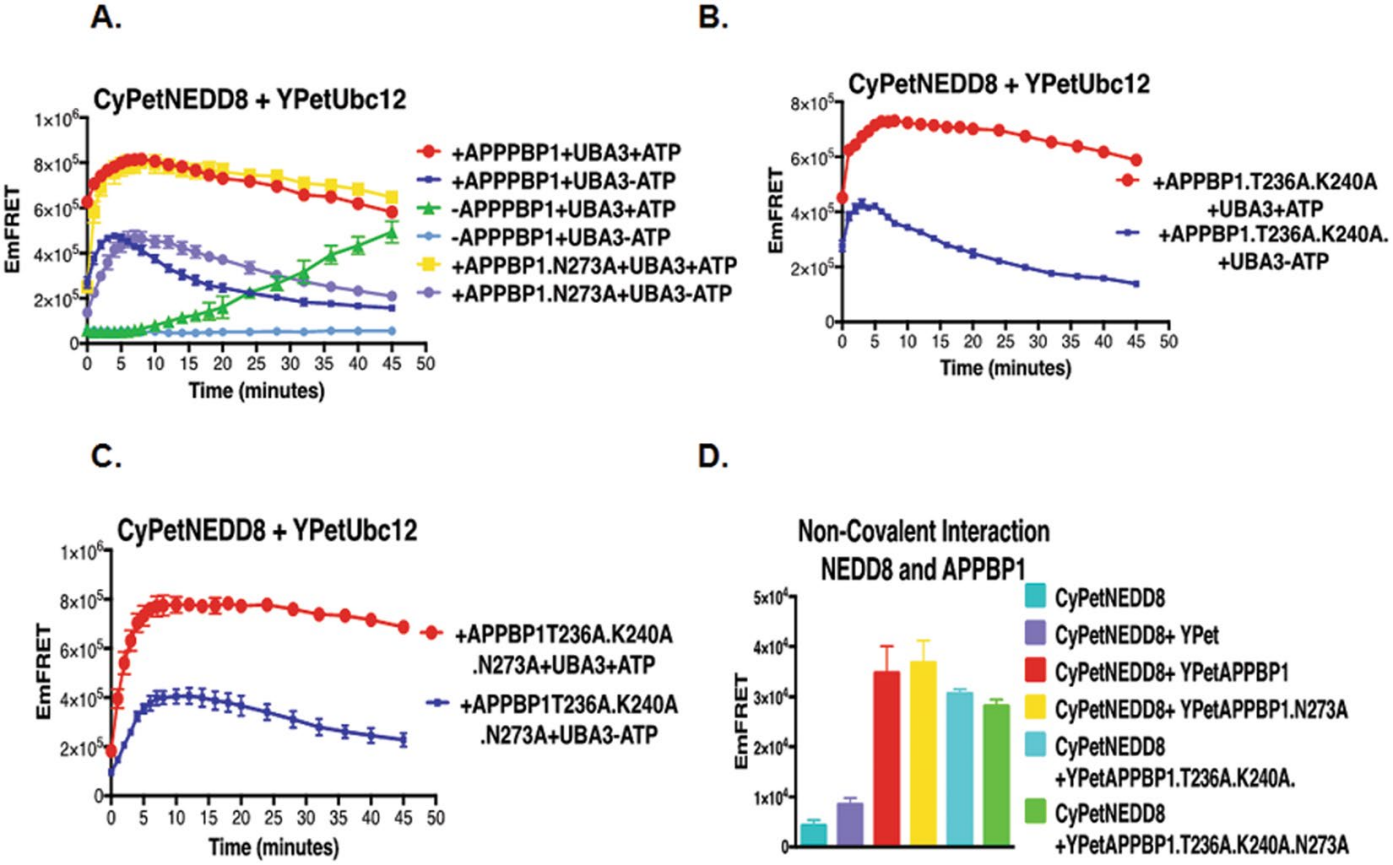
Non-covalent interactions between APPBP1 and NEDD8 are not critical for NEDD8 activation and conjugation. **(A–C**) FRET based protein assay for determining importance of PPBP1 and NEDD8 non-covalent interface for NEDD8 activation and conjugation to Ubc12. (**D**) Effects of mutations in APPBP1 on APPBP1-NEDD8 non-covalent interactions (data are represented as mean +/− SD).

In the E1-NEDD8-ATP complex, both APPBP1 and UBA3 interact with NEDD8. We next investigated the interaction between APPBP1 and NEDD8. Based on the analysis of the interface between APPBP1 and NEDD8, we generated three APPBP1 mutants with increasing numbers of mutations, N273 A, T236A/K240A and T236A/K240A/N273A by using site-directed mutagenesis. The single mutation on APPBP1-N273A did not significantly affect the interaction activity with NEDD8, but both the double (T236A/K240A) and triple (T236A/K240A/N273A) mutants exhibited decreased interactions with NEDD8 (Fig. 3D). Not surprisingly, none of the mutations affected the activation of NEDD8, reaching the same plateau as wild-type APPBP1 (Yellow curve in Fig. 3A–D). However, the effect on the kinetics of activation (at T0) was slightly different: both the double and the triple mutations caused a slight delay of NEDD8 activation (compare the Red and Yellow curves, with the effect of the triple mutation of APPBP1 more pronounced (Fig. 3B,C). The abilities of these mutants to interact with NEDD8 were examined to have no big effect for interactions in our quantitative FRET assay.

**Figure 4:**
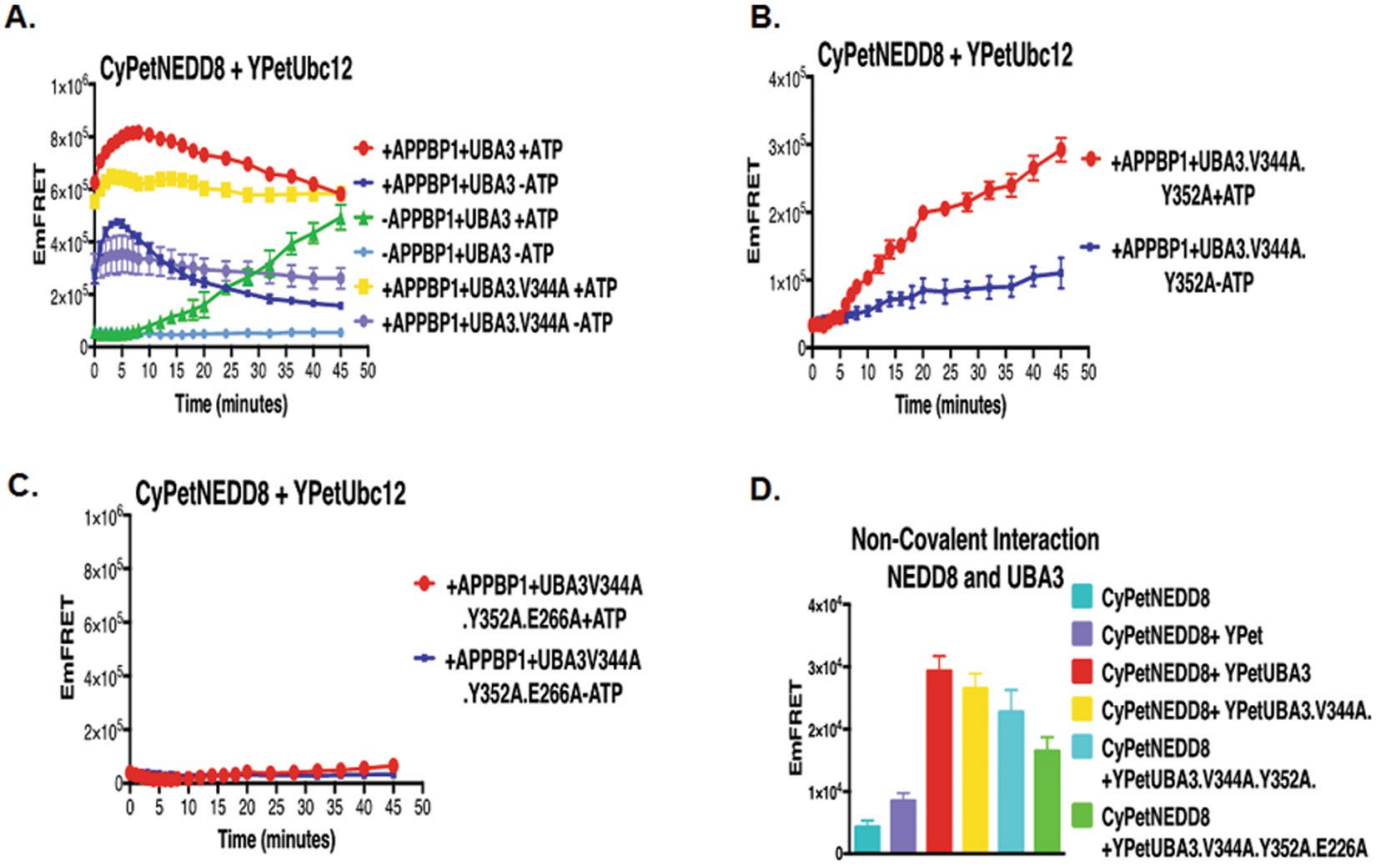
Non-covalent interactions between UBA3 and NEDD8 are critical for NEDD8 activation and conjugation. (**A**) Effects of mutations in UBA3 on UBA3-NEDD8 non-covalent interactions (data are represented as mean +/− SD). (**B**) FRET-based assay of NEDD8 and Uba3V344A mutant in NEED8 activation and conjugation. (**C**) FRET-based assay of NEDD8 and Uba3V344AY352A double mutant in NEDD8 activation and conjugation. (**D**) FRET-based assay of NEDD8 and Uba3V344AY352AE366A triple mutant in NEDD8 activation and conjugation.

*Binding of UBA3 to NEDD8 is required for NEDD8 activation*-The other non-covalent interaction prior activation of NEDD8 is Uba3 and NEDD8^6,13^. We then asked if this interaction is important for NEDD8 activation. The crystal structure of NEDD8-E1 complex demonstrates that the NEDD8 C-terminal tail fits into a groove in UBA3 near the nucleotide-binding pocket^19,21^. Based on the crystal structure and computational mutagenesis, we generated three UBA3 mutants (V344 A, V344A/Y352A, V344A/Y352A/E266A), which exhibited decreased UBA3 interaction with NEDD8, as determined by the quantitative FRET assay (Fig. 4D). The V344A mutation reduced the speed of NEDD8 activation (the yellow and red curves in Fig. 4A), while the V344A/Y352A double mutations markedly repressed NEDD8 activation; the effect of the double mutations was even more profound than that of APPBP1 removal (the yellow and green curves in Fig. 4B). Furthermore, the V344A/Y352A/E266A triple mutations completely prevented NEDD8 activation, even when APPBP1 and ATP were both present (yellow curve in Fig. 4C). Thus, UBA3 and NEDD8 non-covalent interaction prior to adenylation reaction is critical for NEDD8 activation (Fig. 4D).

*Effects of disrupting APPBP1-UBA3 interaction on NEDD8 activation-*The E1 activating enzymes appear to have evolved into a single monomer (e.g., ubiquitin and ISG15) or a heterodimer (e.g., for SUMO and NEDD8). Thus, we speculated, since APPBP1 itself and its interaction with NEDD8/ATP are not essential for NEDD8 activation, APPBP1 interaction with UBA3 should be disposable in the activation of NEDD8 as the result of the evolutionary process. We therefore examined the significance of the interaction between of subunits of E1 heterodimer for NEDD8 activation.

**Figure 5:**
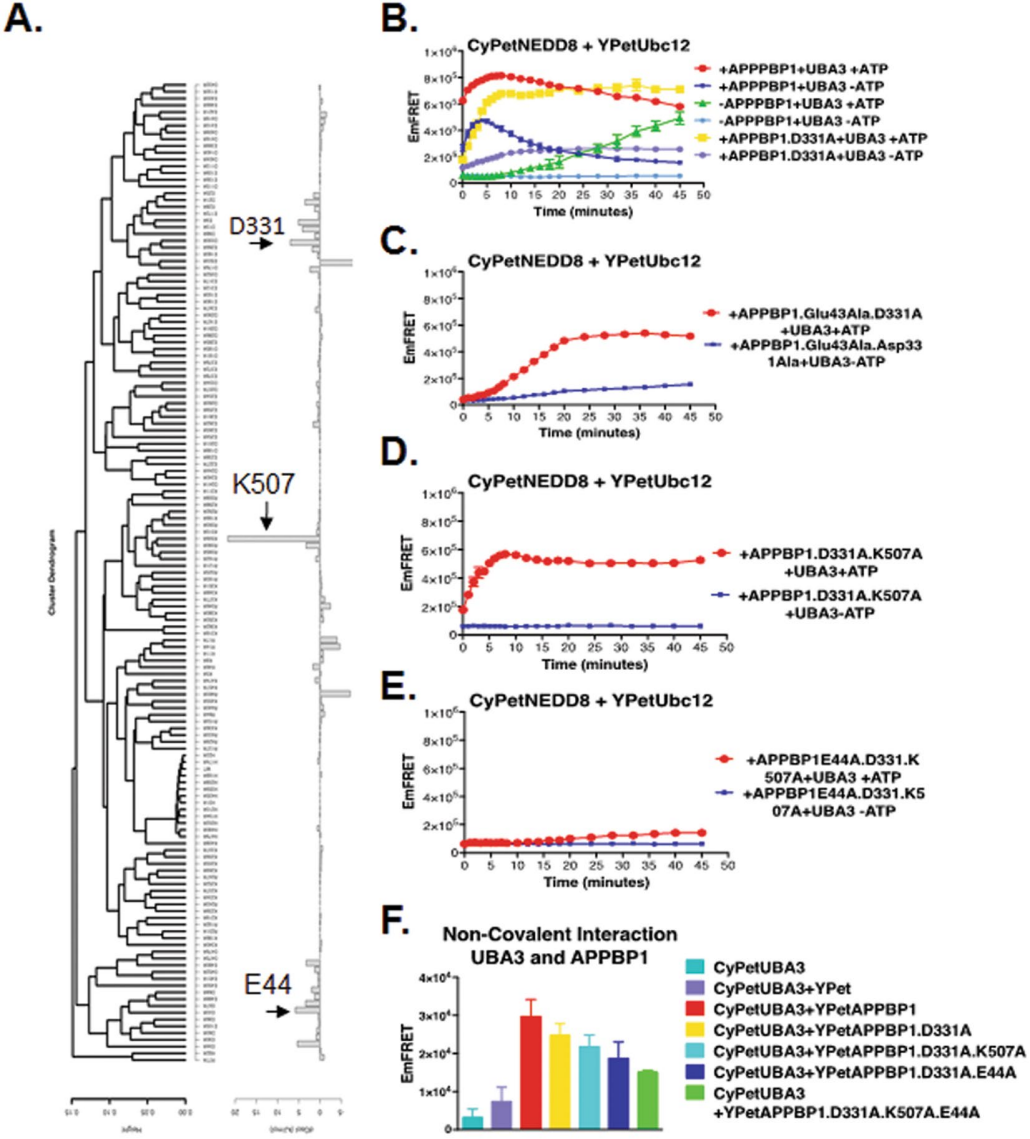
Non-covalent interactions between APPBP1 and UBA3 are important, but required, for NEDD8 activation and conjugation. (**A**) Alanine scan electrostatic clustering and free energies of association. Clustering dendrogram of the alanine scan mutants of APPBP1 using the average weighted difference ESD. Free energy of mutants ordered according to average weighted difference clustering. Non-covalent interactions between APPBP1 and UBA3 are partially critical for NEDD8 activation and conjugation. (**B**) FRET-based assay for interactions of APPBP1 mutants with UBA3 (data are represented as mean +/−SD). (**C**–**E**) FRET-based NEED8 activation assay for APPBP1 mutants disrupting interaction with Uba3.

We first performed a computational mutagenesis study to identify key amino acids for formation of the APPBP1-UBA3 complex. Given that APPBP1 and UBA3 are highly charged, we focused on the effects of ionizable amino acids and electrostatic potentials in association. By using the AESOP computational framework^20–22^, we performed a systematic computational alanine scan analysis, in which we replaced every ionizable amino acid, one at a time, by alanine, followed by calculation and clustering of electrostatic potentials and calculation of electrostatic free energies of association. Figure 5A shows the results of the AESOP analysis, which enabled us to select APPBP1 mutants that were predicted to perturb the APPBP1-UBA3 interface and to assess the effect of the APPBP1-UBA3 complex on the activation of NEDD8. We selected three sites on APPBP1 for mutations: E44A, D331A, and K506A from the calculated electrostatic free energies of association shown in Fig. 5A. These three mutants, E44A, D331A, and K506A, showed the highest perturbation of electrostatic free energies of association, corresponding to loss of binding affinity, and therefore were deemed most important in mediating APPBP1-UBA3 complex formation. Thus, we generated four sets of APPBP1 mutants (i.e., D331A, E44A/D331A, D331A/K506A, and E44A/D331A/K506A) for experimental testing. Consistent with the computational analysis, all the four APPBP1 mutants exhibited decreased interactions with UBA3, as illustrated by the quantitative FRET assay (Fig. 5B). Expectedly, APPBP1 triple mutant (D331A/E44A/K506A) showed the biggest reduction in affinity for UBA3 (green bar in Fig. 5B).

We next examined whether the mutations play any roles in NEDD8 activation. The single mutation D331A reduced NEDD8 activation but did not affect NEDD8 activation and conjugation in long period of time of over 20 min, however it did slow down the initial activation step of the assay (yellow curves in Fig. 5C). The two double mutations D331A/K506A and E44A/D331A dramatically decreased the kinetics of NEDD8 activation and but only moderately reduced NEDD8 activation (yellow curves in Fig. 5D,E), highly reminiscent of the effect of APPBP1 removal/deletion. Intriguingly, the triple mutation D331A/E44A/K506A markedly constrained NEDD8 activation, resulting in a more pronounced effect than the complete removal of APPBP1 (compare Yellow and Green curves in Fig. 5F). The stronger impact of the triple mutations than APPBP1 deletion led us to speculate that the D331A/E44A/K506A mutant may serve as a dominant negative mutant.

## Discussion

**Figure 6:**
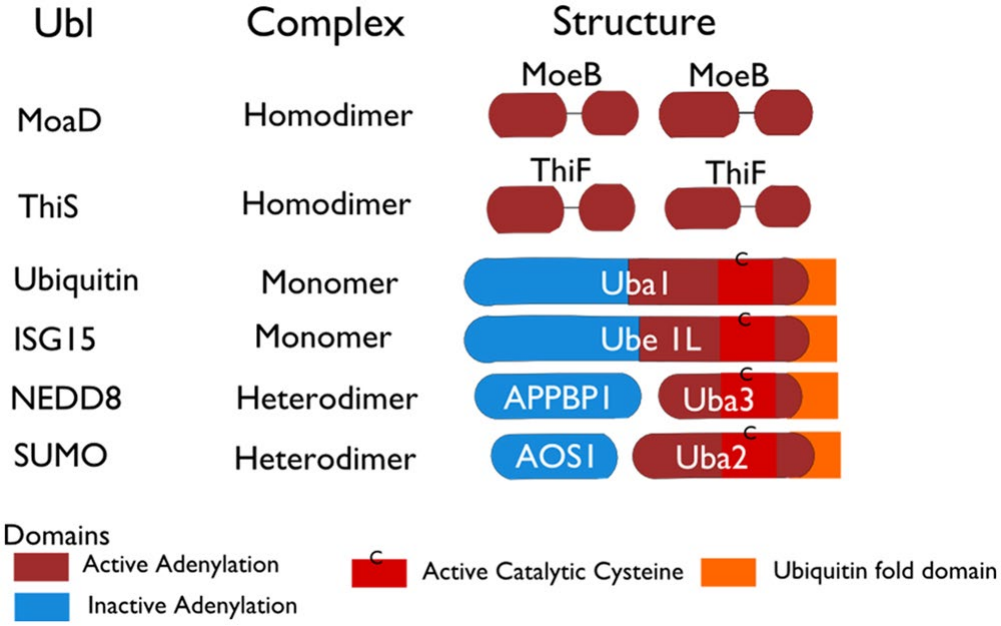
Comparison of activating enzymes for Ubiquitin and Ubl proteins with those for the MoaD and ThiF. The four Ubls whose E1s are most closely related are ubiquitin, SUMO, NEDD8 and ISG15 and their family members. The Ubl E1s are 110–120 kDa and are either single polypeptides or heterodimers of two polypeptides, corresponding to the sequence and structure of MoeB. The E1s of ubiquitin and ISG15 are single polypeptides. In case of heterodimeric E1s (SUMO and NEDD8 cascade), one subunit corresponds to N terminal half of the single-chain E1s and other subunit of the heterodimer corresponds to C-terminal of half. The N- and C-terminal halves of the E1s for ubiquitin, SUMO, NEDD8 and ISG15 are partially homologous to each other, and the region of sequence homology between the two halves is also the region of sequence homology to MoeB and ThiF.

We provide a systematic and detailed investigation of the enzymatic reaction kinetics, reaction intermediates and the landscape of molecular interactions – both covalent and non-covalent – during NEDD8 activation in real time. The covalent interaction means NEDD8 conjugation to UBA3 in the presence of ATP, and the rest interactions are non-covalent interactions, including NEDD8 interaction with UBA3 in the absence of ATP. We demonstrate in a dynamic mode that UBA3 is essential for NEDD8 activation, but APPBP1, the other subunit of the E1 heterodimer, is not required as an activating enzyme, but rather acts as a scaffold protein that accelerates the kinetics of activation. APPBP1 is homologous to the N-terminal domains of Uba1 and AOS1^7,9^. Uba1 is a single-polypeptide E1 activating enzyme for ubiquitin, while heterodimers of AOS1/Uba2 and APPBP1/UBA3 function as the counterpart of Uba1 in SUMO and NEDD8 activations, respectively (Fig. 6). We previously reported that AOS1 and Uba2 are both required for SUMO activation and conjugation to Ubc9 (SUMO E2)^16^. Thus, the ability of UBA3 to activate and conjugate NEDD8 to its E2 conjugating enzyme in the absence of APPBP1, albeit with slower kinetics, is unique to the NEDD8 conjugation cascade (Fig. 1). It was shown in an earlier report that ECR1 (the UBA3 counterpart in plants) can produce the NEDD8 adenylate intermediate in the absence of AXR1 (the APPBP1 counterpart in plants)^22^. However, the roles and molecular contributions of AXR1 to NEDD8 activation are not understood. Here we elucidate, for the first time, the distinct roles of APPBP1 and UBA3 in the NEDDylation cascade in dynamic mode. Our discoveries also provide valuable insights into the molecular evolutionary pathway of Ubl conjugating cascades.

Interestingly, in spite of disposability of APPBP1 in the NEDD8 activation, the APPBP1 triple mutant disrupting the interaction with Uba3 act as dominant negative to completely abolish the activation of NEDD8 by Uba3. The interaction between APPBP1 and UBA3 may be important for stabilization of the APPBP1-UBA3-NEDD8 complex. In the NEDD8-E2 conjugation assay, mutations of amino acids on APPBP1 interface interacting with UBA3 mutated (APPBP1 mutations D331A, E44A, K506A) result in unfavorable interactions in their local microenvironment at the interface with UBA3 (Supplement Fig. 2). The E44 of APPBP1 interacts with K56 of UBA3, forming a salt bridge (distance is ~3.5 Å); but when K56 loses it salt bridge partner because of E44 mutation to alanine, it finds itself in an unfavorable solvent-deprived environment at the APPBP1-UBA3 interface, with the possibility of an additional unfavorable interaction with APPBP1’s H491 (Supplement Fig. 2). Similarly, the other two mutations of APPBP1, K507A and D331A, may cause perturbations in the local microenvironment of the solvent-deprived association interface because they lead to unpaired charged UBA3 residues D326 and R223, respectively (Supplement Fig. 2). The side chains of these residues are expected to either assume new charge-charge or hydrogen bonding interactions or become solvent exposed; in either case, they are likely to introduce local conformational changes, thus affecting the stability of the APPBP1-UBA3-NEDD8 complex. As a result, single or double mutations of the three amino acids slows down the kinetics of the activation and conjugation reaction, likely due to compromised recruitment of UBA3 and consequent decrease in the accessibility of UBA3 (the activation enzyme) to NEDD8 (the substrate) (Fig. 4E). Interestingly, triple mutations of the three residues of APPBP1 (D331, K507, E44) markedly decreases NEDD8 activation, causing a more pronounced effect than the complete removal of APPBP1, We speculate that the D331A/E44A/K506A mutant may serve as a dominant negative mutant that can no longer interact with UBA3 and instead competes for NEDD8 binding with UBA3, thus limiting UBA3-NEDD8 interaction and catalytic reaction.

With the discovery of dispensable of E1 heterodimer, APPBP1, in the activation NEDD8, we speculate that the potential molecular evolution process of E1 activating enzymes for Ubls and bacterial counterparts, MoaD and ThiS (Fig. 6). All the E1 enzymes shares a homology domain (Red block in Fig. 6). But the E1 activating enzyme in eukaryotes share another homology domain (Blue block in Fig. 6). We can speculate that the evolution process evolving from simple catalytic subunit in bacterial to a heterodimer enzyme with two subunits for NEDD8 and SUMO, one as catalytic and one as scaffold, and then one subunit E1 enzyme with one fusion protein harboring both catalytic and scaffold domain for Ubiquitin and ISGF15 (Fig. 6). This speculation from molecular mechanism elucidation provides a new aspect not only in evolution but important rational in drug design and discovery aiming to disrupt protein-protein interactions in the Ubl pathways.

## Electronic supplementary material

Supplementary Figures
