# Supplementary material for: Dissecting Distinct Roles of NEDDylation E1 Ligase Heterodimer APPBP1 and UBA3 Reveals Potential Evolution Process for Activation of Ubiquitin-related Pathways
Source: Sci Rep. 2018 Jul 4;8:10108. doi: 10.1038/s41598-018-28214-2 (PMC6031683; doi:10.1038/s41598-018-28214-2)

**Dissecting Distinct Roles of NEDDylation E1 Ligase Heterodimer APPBP1 and UBA3 Reveals Potential Evolution Process for Activation of Ubiquitin-related Pathways**

Harbani Kaur Malik-Chaudhry^a^, Zied Gaieb^a^, Amanda Saavedra^a^, Michael Reyes^a^, , Raphael Kung^a^, Frank Le^a^, Dimitrios Morikis^a,b^ and Jiayu Liao^a,b^

^a^Department of Bioengineering, Center for Bioengineering Research, Bourns College of Engineering; ^b^Institute for Integrative Genome Biology, University of California at Riverside, 900 University Avenue, Riverside, CA 92521

**Supplement Method**

*Quantitative FRET analysis.* A highly sensitive FRET pair, CyPet and YPet, was used because of their strong fluorescent signals (32). CyPet and YPet have maximum excitation wavelengths of 441 and 475 nm and maximum emission wavelengths of 475 and 530 nm, respectively. However, the FRET signal at an emission wavelength of 530 nm consists of three fluorescent signals: the absolute FRET signal (Em_FRET_) and the direct emissions of CyPet (CyPet_[direct]_ and YPet_[direct]_ ) when excited at the CyPet excitation wavelength 414 nm (Fig.1B). To monitor the molecular interaction events quantitatively, we designed a method of cross-wavelength correlation coefficiency for fluorescent signal elucidation (31). In brief, two correlation coefficients, α and **β**, for the donor CyPet and acceptor YPet, respectively, were introduced. The α is defined as the ratio of CyPet emission signal at 530 nm (FL_DA_) to emission signal at 475 nm (FL_DD_) when excited at 414 nm (Fig.1C and D). The β is defined as ratio of YPet emission signal at 530 nm when excited at 475 nm (FL_AA_) vs. 414 nm (FL_AD_) (Fig.1D and E). The direct emissions of CyPet and YPet are determined by the products of α*FL_DD_ and β*FL_AA_, respectively. The α and β values were calculated as 0.31 and 0.026, respectively, using CyPet-NEDD8 and YPet-Ubc12 solutions. Therefore, the absolute FRET signal (Em_FRET_) could be determined by subtracting the CyPet and YPet direct emissions from the total fluorescent signals (Fig.1D). We applied the quantitative FRET assay to examination of intermediate formation and the dynamics of NEDD8 conjugation.

**Supplement Figure 1. Design of FRET assay and quantitative FRET measurement.** (A) CyPet-tagged Nedd8 interaction YPet-tagged NEDD8 ligases and substrate. (B) Dissection of emission spectra from a mixture of CyPet- and YPet-tagged protein mixture. Fluorescent emission at acceptor, YPet, wavelength (530 nm; FL_DA_) can be divided into three sections: FRET emission from YPet, direct emission of donor, CyPet, and direct emission of acceptor, YPet. (C) Donor emission at 475 nm (FL_DD_) and 530 nm (FL_DA_) when excited at 414 nm. (D) Acceptor emissions at 530 nm when excited at 414 nm (FL_AD_) or 475 nm (FL_AA_) (E) Definition of Em_FRET_.


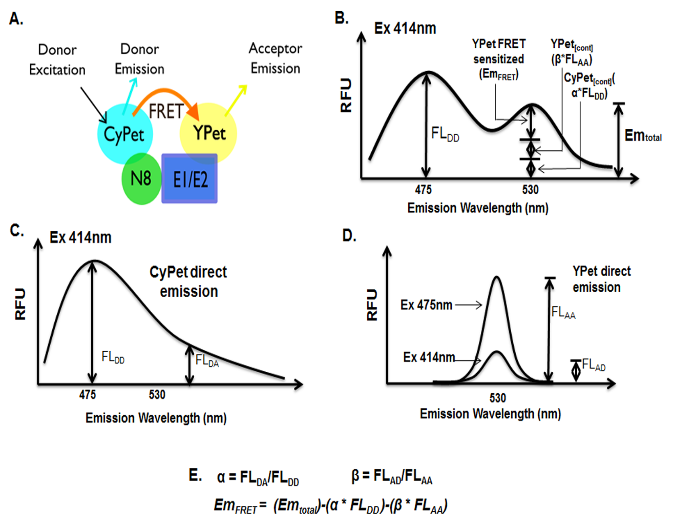


**Supplement Figure 2. Close-up view of APPBP1 and UBA3 interface.** (PDB ID: 1R4N), UBA3 shown in red, APPBP1 in blue, NEDD8 in green, ATP in magenta, APPBP1 residue in cyan, UBA3 residue in orange, possible unfavorable interaction residue in grey. (A) Possible interactions interface between APPBP1’s E44 and UBA3’s K65. In the E44A mutation, UBA3’s K56 and APPBP1’s H491 have unfavorable interactions. (B) Possible interactions interface between APPBP1’s K507 and UBA3’s D326. In the K507A mutation, UBA3’s D326 and APPBP1’s E504 have unfavorable interactions. (C) Possible interactions interface between APPBP1’s D331 and UBA3’s R223. In the D331A mutation, UBA3’s R223 and UBA3’s H227 have unfavorable interactions.


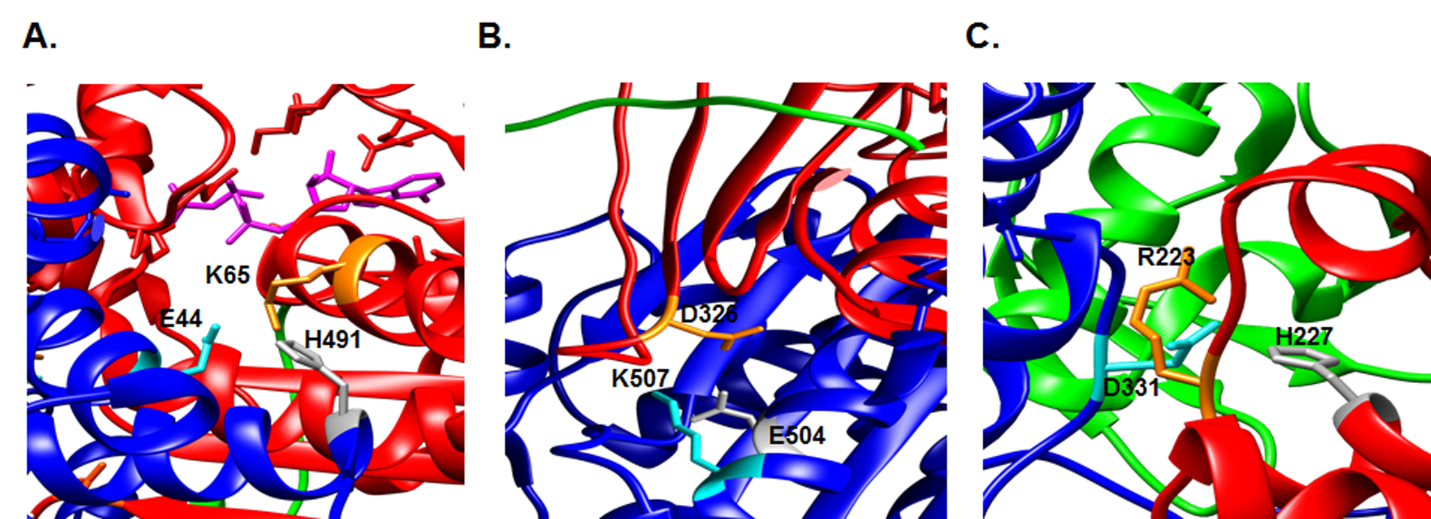


**Supplment Figure 3. Different exposure of NEDDylaiton assay in Figure 2B.**


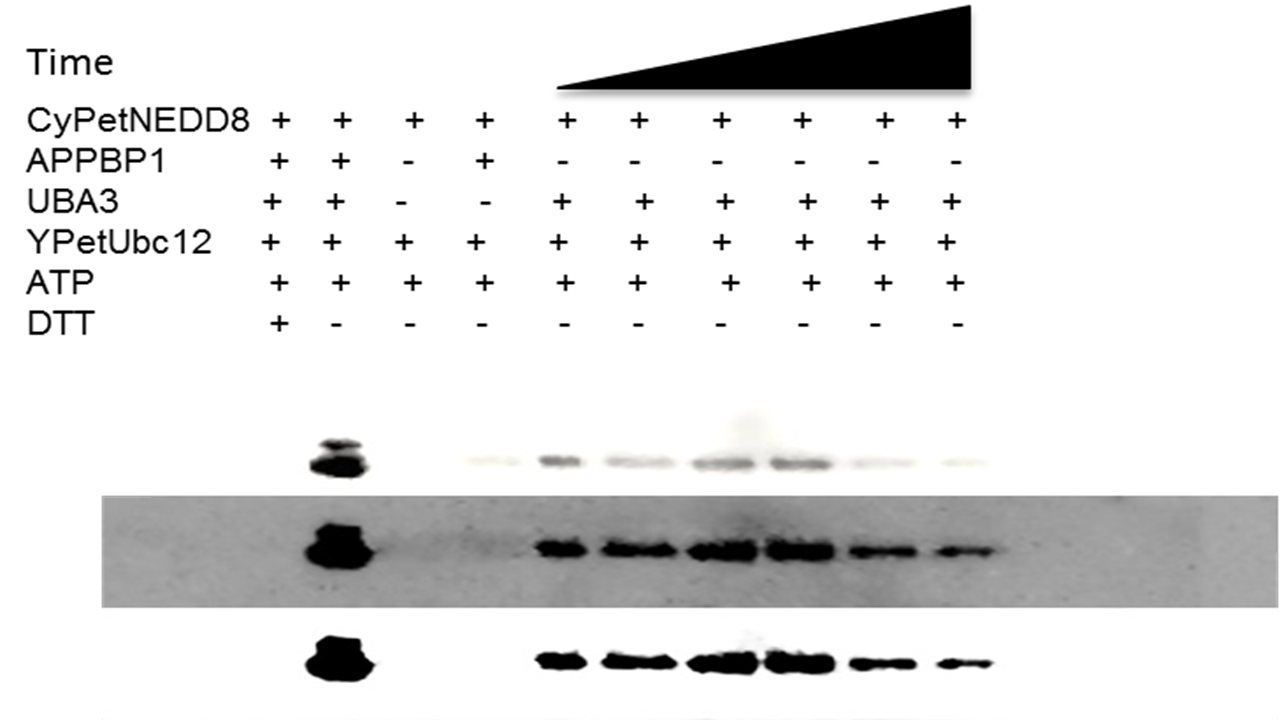

Supplement: Supplementary file 1 — Supplementary Figures [file 41598_2018_28214_MOESM1_ESM.docx]
